# Xanthogranulomatous Orchitis With Testicular Atrophy in a Case of Chronic Pyocele: A Rare Case

**DOI:** 10.7759/cureus.58325

**Published:** 2024-04-15

**Authors:** Sagarika S Bhole, Gowardhan Dare, Mohd Yunus Shah, Aashima A Prakashe

**Affiliations:** 1 Surgery, NKP Salve Institute of Medical Sciences, Nagpur, IND

**Keywords:** testicular atrophy, scrotal swelling, scrotal pyocele, xanthogranulomatous orchitis, xanthogranulomatous inflammation

## Abstract

Xanthogranulomatous orchitis (XGO) is a benign non-inflammatory condition predominantly affecting the testicular tissue and characterized by the infiltration of lipid-laden macrophages leading to substantial tissue damage. We present the case of an 80-year-old gentleman with chronic pyocele and concurrent testicular atrophy secondary to XGO, a seldom-reported manifestation in the testicular milieu. The patient presented with a protracted history of left-sided scrotal swelling and underwent left orchidectomy subsequent to preoperative diagnosis via ultrasonography. Intraoperative exploration revealed the presence of purulent fluid, and histopathological analysis confirmed characteristic features of XGO, including seminiferous tubule destruction and infiltration of fibroconnective tissue by histiocytes and dilated blood vessels. Differential diagnosis with testicular neoplasms posed a challenge, accentuating the pivotal role of histopathological scrutiny in achieving precise diagnosis. Orchidectomy remains the cornerstone of treatment for XGO. This case underscores the imperative of considering XGO in the diagnostic spectrum of testicular masses and the indispensable role of histopathology in confirming the diagnosis and guiding optimal therapeutic interventions.

## Introduction

Xanthogranulomatous inflammation is a rare benign etiology characterized by the infiltration of lipid-laden macrophages with associated tissue damage [1]. Xanthogranulomatous inflammation commonly affects the kidneys accounting for 0.6% to 1.4% of renal infections. It is also shown to affect the gallbladder, liver, appendix, ovaries, vagina, bones, and urinary bladder [2]. Its incidence in the testis is very rare. When it affects the testis, it is termed as xanthogranulomatous orchitis (XGO). XGO is a rare benign illness of the testes characterized by the infiltration of lipid-laden macrophages that replace the dying testicular tissue [3].

We report the case of an 80-year-old male who presented with xanthogranulomatous ­inflammation with testicular atrophy and chronic pyocele of the scrotum.

## Case presentation

**Figure 1:**
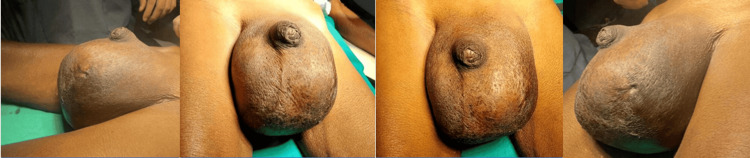
Preoperative view of swelling over the left testis.

An 80-year-old male patient reported a 40-year history of left-sided scrotal swelling (Figure 1), which had been associated with pain for the past month. The swelling had gradually increased in size over time. Additionally, the patient had undergone fluid aspiration from the scrotum 10 years ago at a small clinic. He denied experiencing urinary symptoms, urethral discharge, or fever and had no comorbidities. On clinical examination of the right testis, it was palpable and non-tender and was found to be normal. The left testis could not be palpated separately, and the respective scrotum was firm with a non-tender swelling of 25 × 15 cm. Palpation above the swelling was possible. Scrotal rugosities were absent over the swelling. A scar from a previous aspiration was visible on the left scrotum. The transillumination test was negative.

Ultrasonography and Doppler of the scrotum revealed gross free fluid with multiple dense echoes in the left tunica vaginalis sac with left atrophic testis suggestive of chronic left-sided hydrocele. The decision was taken to post the patient for left-sided sac eversion with left-sided orchidectomy.

**Figure 2:**
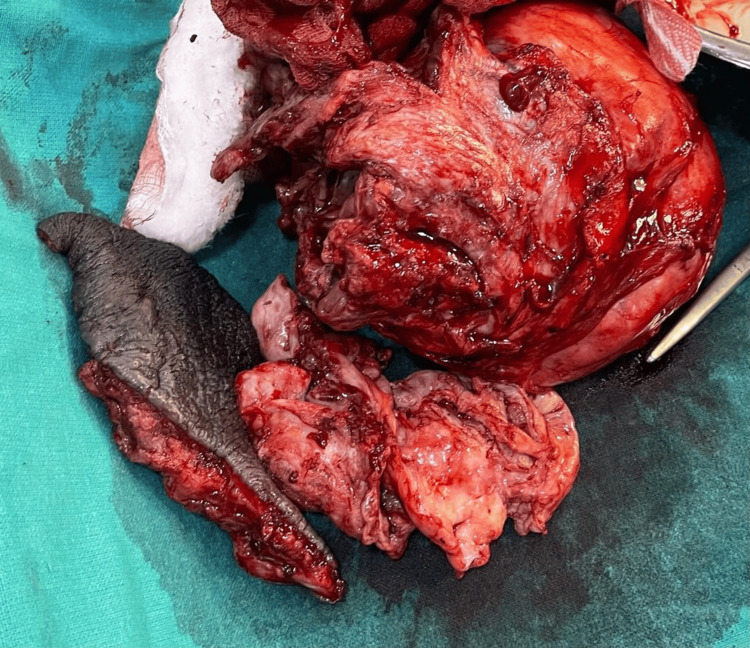
Specimen after orchidectomy and partial scrotectomy consisting of left-sided atrophic testis and excessive scrotal skin.

**Figure 3:**
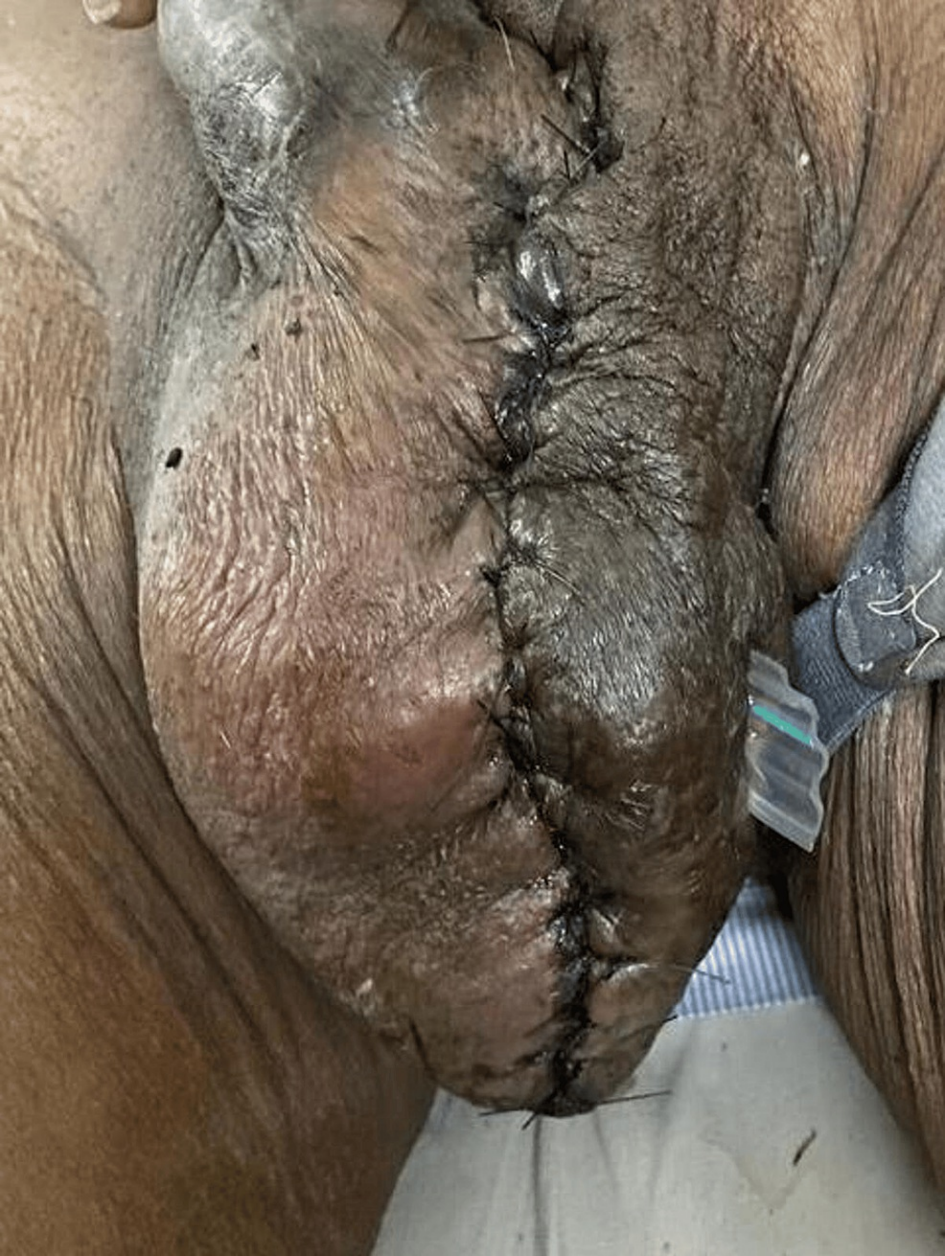
Postoperative photos of the wound with a corrugated rubber drain in situ.

Intraoperatively, on incising the tunica vaginalis, approximately 160 mL of pus was aspirated. There was evidence of multiple septations, and the left testis was atrophied. Left-sided orchidectomy with partial scrotectomy was performed under spinal anesthesia (Figure 2). A corrugated rubber drain was placed in situ (Figure 3). The postoperative course was uneventful, and the patient was discharged on postoperative day 10 after suture removal.

On recall after two months, the healing was uneventful and the wound had healed adequately with no evidence of wound gape or discharge.

**Figure 4:**
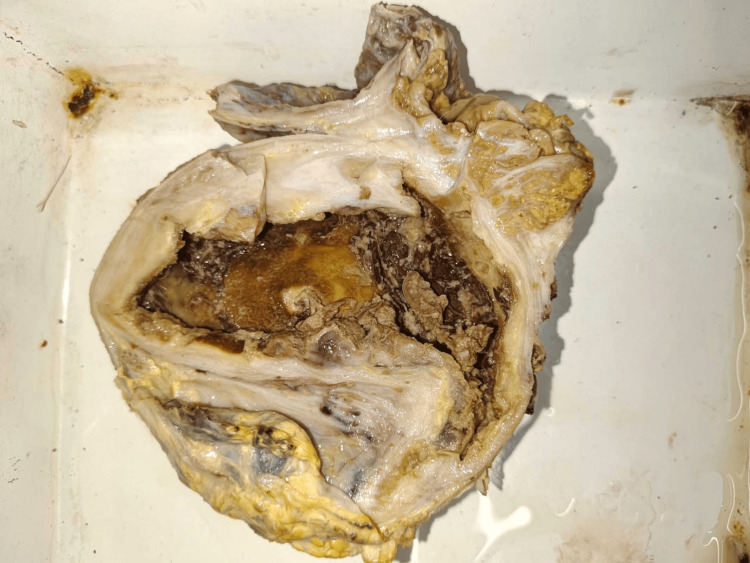
Gross pathology showing the testis appearing necrotic and atrophic, surrounded by dense fibrotic tissue and yellowish-white deposits in the paratesticular area.

On gross pathology, the testis appears necrotic and atrophic, surrounded by dense fibrotic tissue and yellowish-white deposits in the paratesticular area (Figure 4).

Microscopic examination

The histopathological examination revealed mostly fibroconnective tissue showing many congested and dilated blood vessels, capillaries, and adipose tissue. A few areas showed sheets of macrophages, histiocytes, and focal lymphocytic collections. Moreover, areas showing myxoid changes, microcalcifications, hemorrhage, and necrosis were also seen.

**Figure 5:**
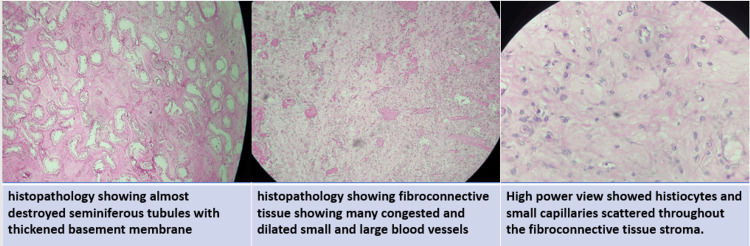
Histopathological findings.

Sections from the testicular area revealed a few seminiferous tubules with a thickened basement membrane lined by simple cuboidal epithelial cells with intervening connective tissue showing fibrosis (Figure 5).

## Discussion

A rare inflammatory disease that affects the testis, XGO is characterized by the destruction of non-neoplastic tissue and the possibility of mass-like lesions that frequently resemble cancers [4]. Similar to its manifestation in other organs such as the kidney, gallbladder, and appendix, the primary causes are believed to be obstructive processes and chronic infections. Similarly, urinary tract infections and spermatic cord blockage are important components in the pathophysiology of the testis [5,6].

Because of its symptoms and radiological features that closely resemble those of other testicular tumors, diagnosing XGO before surgery can be challenging. Before diagnosing XGO, it is necessary to rule out the differential diagnoses, which include malakoplakia, Rosai-Dorfman disease, and unusual infections. Large histiocytes with eosinophilic cytoplasm (Von Hansemann cells) and calcified phagosomes (Michaelis-Gutmann bodies) are what histological malakoplakia is known for. Rosai-Dorfman disease is a histiocytosis that does not include Langerhans cells and typically results in painless lymphadenopathy. Nonetheless, extranodal involvement is present in 40% of cases, with the testis involved in many of them. Histiocytes stain positively for S100 and exhibit emperipolesis under the microscope. Certain stains, such as Grocott and Periodic acid-Schiff stains for fungal organisms, Ziehl-Neelsen for *Mycobacterium tuberculosis* and Wade-Fite for *Mycobacterium leprae*, and Ziehl-Neelsen for Mycobacterium tuberculosis, can be used to visualize atypical infections, especially those involving mycoplasma or fungal organisms, which can cause granulomatous reactions. In our case, the drained fluid and histopathology did not reveal signs of tuberculosis, which was earlier kept as a differential diagnosis [4,7,8].

Before surgery, elevated levels of serum tumor biomarkers, such as placental alkaline phosphatase, CD117, and OCT3/4, can offer a preliminary diagnosis. It can be difficult to distinguish between the two illnesses because these indicators may stay within normal levels in some forms of testicular tumors. As a result, histological investigation of the tissue sample taken during surgery is frequently crucial to an appropriate diagnosis [2,4,7,8].

Orchidectomy is the gold standard for XGO. On a microscopic level, XGO has groups of foamy histiocytes and other inflammatory cells that help destroy the testicular tissue [7,8].

## Conclusions

XGO is an infrequent condition, as evident from the paucity of the literature. Clinicians must be aware of the potential mimickers before planning any radical operative procedure, whereas pathologists must be aware of its notoriety in masking an underlying testicular tumor. A comprehensive workup involving adequate history, thorough clinical examination, radiological investigation, and serum biochemistry for tumor biomarkers is important to provide clues; however, pathological examination is imperative for a conclusive diagnosis and to rule out any germ cell tumor. This case is a reminder of the rare differential diagnosis for testicular pathology and adds another example to the medical literature.
